# Qualitative Purpose Profiles of Chinese Student Teachers

**DOI:** 10.3390/ijerph191912745

**Published:** 2022-10-05

**Authors:** Fei Jiang, Timothy S. Reilly, Jenni Menon Mariano

**Affiliations:** 1 Center for Ideological and Political Education, Northeast Normal University, Changchun 130024, China; 2 Department of Psychology, Ave Maria University, Ave Maria, FL 34142, USA; 3 Department of Educational and Psychological Studies, University of South Florida, Tampa, FL 33620, USA

**Keywords:** purpose profile, Chinese, student teachers

## Abstract

Teachers are well positioned to help students cultivate their purpose in life, which is an asset that is associated with optimal development. Teachers must also have a grasp on their own sense of purpose, especially during times of intense social pressure and change, when the capability to sustain and support worthy aims may impart personal resilience and contribute to the social good. To train educators who have this capability, it is therefore vital for teacher education programs to in turn understand their own students’ individualized purpose statuses. Using a qualitative person-centered approach, the current study identified purpose profiles of teacher education candidates in China as part of a larger multinational study. Three hundred and thirty-one participants wrote answers to questions about the content and fulfillment of their purpose in life, and statements were reliably coded for how specifically the respondents referenced their purpose, and for whether their purpose aimed to benefit others. A consensual qualitative research approach then identified four purpose profiles: beyond-the-self purpose, self-oriented life goal, daydreamer and purposeless. The meaning of these profiles and their significance for cultivating purpose among China’s teachers are discussed.

## 1. Introduction

Purpose provides people with guidance amidst the difficulties of life, as purposeful individuals are motivated to fulfill themselves through their pursuits and in this to support loved ones and society [1] (p. 8, 149) and to accomplish something meaningful to the world [2] (p. 12). It is generally thought that the inclination to seek purpose and the benefits purpose brings are universal to human beings; yet, the discovery of one’s purpose, its content, and its pathways are unique and personal, able to be fulfilled by the individual alone [2] (p. 12). As such, while variable-centered methods identify what is common among individuals, person-centered approaches such as qualitative profile analysis have the potential to capture and describe the uniqueness and diversity of individuals’ purpose experiences. Qualitative analyses can provide rich descriptions about individuals’ purpose that are nuanced and less easily captured by quantitative measures, which can be helpful for educators to understand the unique developmental and learning needs of their students. In the tradition of identifying both unique and universal features of purpose, recent research has focused on the important role of purpose in different cultural settings [3,4,5,6,7,8] (pp. 148–162; pp. 565–581; pp. 921–937; pp. 231–244; pp. 348–377; pp. 16–33), and a few studies have examined the qualitative purpose profiles of younger people in different contexts [9,10,11,12,13] (pp. 35–44; p. 77; pp. 715–733; pp. 37–41; pp. 143–159). One qualitative approach initially found five profiles with regard to young people’s purposeful pursuits [10] (pp. 59–61). Subsequently, other scholars have a replicated or extended this approach with other populations, sometimes further specifying and multiplying the initial profiles [5,13,14,15,16,17,18,19,20,21,22] (pp. 921–937; pp. 143–159; pp. 245–257; p. 38; pp.101–109; pp. 133–145; pp. 186–199; pp. 539–558; pp. 186–199; pp. 175–185; pp. 272–282).

In this article, we examine the purpose profiles and perspectives of Chinese student teachers, seeking to understand how they discuss their purpose in life. Teachers play a significant role in students’ purpose formation and support their students’ purpose development. Given this, how teachers view their own purpose is of extreme importance. Student teachers are in a phase of their occupational trajectory that provides a special opportunity in understanding purpose. They are educated and trained at the same time as they engage with K-12 schools to develop their capacity as educators. In this sense, there is still room for educational guidance for these student teachers, especially in preparing them to guide their future students’ purpose development. Though researchers have long noted the significant role that purpose may play in life and given substantial attention to factors that may contribute to the development of purpose, insufficient attention has been given to this special group of student teachers and how they articulate their purpose in life. Accordingly, the aim of this study is to fill the gap of understanding purpose profiles among student teachers and to provide insight for future teacher education initiatives that hope to cultivate actively developed altruistic purposes among student teachers.

### 1.1. The Global Study of Purpose

Scholars emphasize three key elements of purpose in life: (1) commitment [10,23,24] (p. 2; pp. 78–109; pp. 119–128), (2) goal-directedness [25,26] (pp. 242–251; pp. 13–39), and (3) personal meaningfulness [25,27] (pp. 242–251; pp. 1–28). For many thinkers, however, purpose can be viewed as a ‘moral beacon’, not just as a life goal. By acting as a beacon toward the good, purpose guides individuals to commit to and engage in prosocial, generative behaviors across their lifespans [28] (pp. 526–531). Purpose differs from related concepts such as values and meaning in life largely in its emphasis on influencing the world, rather than gratifying oneself or achieving self-oriented goals [3,29] (pp. 148–162; p. 2106). Purpose in life therefore motivates people to pursue a stable intention so as to achieve personally meaningful goals while contributing to the world [10] (p. 26). A strong and enduring purpose is often an individuals’ highest goal [6] (pp. 231–244), and pursuing this goal can foster perseverance and resilience in the face of negative emotions [30] (p. 133). Purpose has been associated with psychological well-being [31,32] (pp. 500–510; pp. 337–345) and physical well-being, as well as identity formation [1] (pp. 8, 149). In contrast, youth lacking purpose are more likely to be involved in antisocial and risky behaviors such as abusing drugs and alcohol [33] (pp. 33–52) and are at greater risk of death [34]. 

China in particular has long traditions of valuing the guiding and inspiring role that purpose can play in individuals’ lives. In the Chinese context, purpose can be understood as a spiritual pillar and driving force of life, determining one’s direction and life path [35] (pp. 43–45). Purpose, in China, can be characterized as seeking a more perfect future by means of activity which is valuable both to society and to oneself [36] (p. 149). Based on the realistic possibility of refinement of the specific aims individuals pursue as they seek to realize their purpose, purpose is seen as a human-specific spiritual phenomenon [35] (pp. 43–45). As such, an ideal purpose conforms to the needs of the current social circumstances and historical needs of society. Purpose can also be seen as the valid and rational anticipation or imagination of the future, representing people’s worldview, values and outlook on life, and reflected in their striving goals. In this sense, purpose is the core of people’s spiritual life [37] (p. 1). Thus, scholars in China have described similar fundamental ideas about purpose to those of the rest of the world, despite their distinctive cultural emphases and linguistic expressions. They understand adolescents around the world as sharing common cognitive processes, which may shape the relation of their lives to the world [38] (p. 5). Scholars worldwide agree that an active life purpose has the following characteristics: a clear aim with future orientation, participation in particular behaviors and great efforts made not only for personal achievement but for social development [4,39] (pp. 565–581; pp. 110–127).

### 1.2. Qualitative Purpose Profiles

Not all youth are purposeful, and while purpose can be conceived as a matter of degree and assessed from a variable-centered perspective, categorical person-centered approaches are useful for illumining an individual-specific understanding of purpose and life goal pursuit [40]. Studies that give attention to this approach often operationalize purpose and related categories according to three dimensions: (1) intention, (2) engagement, and (3) beyond-the-self or prosocial pursuits [13] (pp. 143–159). Damon [10] (pp. 59–61), for instance, drew on a national sample of young people in the United States and divided them into five categories based on these dimensions: the disengaged, the non-purposeful, the dreamers, the dabblers, and the purposeful. Follow-up research [19] (pp. 186–199) elaborated these categories further. Dreamers were understood as having a strong sense of meaning and beyond-the-self intention, but without substantial or sustained engagement with that intention. Dabblers, in contrast, were engaged in potentially purposeful activity, with prosocial or beyond-the-self consequences, but lacked strong intentionality about this activity. The non-purposeful, which included youth pursuing self-oriented goals [19] (pp. 186–199) were intentional and engaged, but directed their efforts not toward the world beyond-the-self or toward prosocial goals, but primarily toward their own self-advancement or satisfaction. Purposeful youth were those who coordinate all three dimensions, intentionally engaging with their beyond-the-self and/or prosocial goals. In the study under discussion [19] (pp. 186–199), it was also suggested that one might further classify self-oriented dreamers as those who have strong self-oriented desires that they are not consistently pursuing, while self-oriented dabblers are those who are actively engaged in pursuing seemingly satisfying and self-advancing pursuits, but who are not strongly intentional about doing so. Finally, the disengaged pose what is likely the greatest challenge for parents and educators, as they showed little evidence of strong intentions or purposeful activity. Beyond-the-self dreaming, self-oriented goal pursuit, and beyond-the-self dabbling can be considered precursor forms of purpose, based on the level of intention, engagement, and type of motivation [19,41], as in each case, youth demonstrated key components of purpose. Further, this study found that older youth, especially those pursuing careers in helping professions (e.g., teaching and medicine) were more likely to be identified as purposeful than younger youth or those entering other professional fields such as business [19] (pp. 186–199). 

Drawing on these categories, scholars have examined the sources of support that young people experience in pursuing their purposes [5,7] (pp. 921–937; pp. 348–377). These categories have also been related to differences in language use, with purposeful youth expressing more positive coping, generosity, gratitude, and empathy than others [42] (pp. 1–24). Further, in analysis of a separate sample of emerging adults in the United States [11] (pp. 715–733), Glanzer and colleagues identified similar categories: the Directionless, the Achievers, the Relationalists (who identified their purpose as relating to others or to personal ideals), the Religious, and Change the World Transformers. These youth pursued purposeful aims at a rate similar to that of those in Damon’s study [10] (pp. 59–61), with somewhere between 20% and 30% of the sample pursuing prosocial and beyond-the-self life-goals.

Less evidence is available regarding Damon’s categories in China. Nonetheless, as described above, there is reason to believe that Chinese culture holds similar ideas about purpose to those found in other studies. Further, Chinese college students’ anticipated future needs structure relates closely to purpose, since people’s future needs are reflected in their intentions and central goals [43] (p. 185). An empirical study among 529 Chinese college students discovered four categories of needs structures [12] (pp. 37–41). For those students belonging to the development-type (~87% of the sample), the most important needs were knowledge, friendship, respect and achievements, in that order. Conflict-type students (~8% of the sample) identified friendship, achievements, material, and contribution needs, again in that order, as their most important future needs. Enjoyment-type (~4% of the sample) students valued needs in this order: material, survival, maintenance, and friendship. Finally, consecration-type (2%) students prioritized aesthetic pursuits most highly, followed by helping others, power, and contribution, in that order. Another 20-year longitudinal study conducted in four waves among 4620 Chinese college students between 1981 and 2000 classified students’ needs into three categories based on the relative centrality of different needs [44] (p. 2). The first category comprised students who prioritized needs for growth (i.e., abundant material success, aesthetic appreciation, recreation, love, physical exercise and friendship needs). The second category included students who identify most with needs for talent (i.e., needs for professional learning, pursuit of truth, honor, self-respect and moral cultivation). The final category included students who most value needs for obedience [44] (p. 2).

### 1.3. Research Strategy and Questions

Given the multiple dimensions and subjective nature of purpose in life, a range of methodological approaches and data collection strategies have been adopted in the study of purpose. These include surveys, interviews, diary studies, and historical document reviews [30,45] (pp. 21–42; pp. 31–44). Among the qualitative approaches, clinical style interviews and short answer statements are commonly used to assess categorical characteristics and forms of purpose and life goals [7,20,42] (pp. 348–377; pp. 175–185; pp. 1–24) alongside informant reports [46] (pp. 281–293). In order to examine the presence and characteristics of purpose profiles among Chinese student teachers, we adopted a self-reported open-ended statement questionnaire based within the context of a global study on the impact of collegiate field experiences on young people’s purpose in life. Our research questions focus on the subjects’ purpose content (i.e., what goal(s) they intend to pursue) and engagements (i.e., what actions they take relating to their purpose). Using a qualitative coding and sorting method we asked whether Chinese teacher education candidates could be portrayed according to descriptive profiles based on the extent to which they elaborate on their self-reported purpose in life.

## 2. Materials and Methods

### 2.1. Participants and Procedure

Three hundred and forty-eight student teachers were surveyed from one normal university (teachers’ university) in China. A total of 331 (male 19.4%) valid questionnaires were collected with participants aged from 19 to 26 years old (*M =* 20.22; *SD* = 1.12), indicating a valid response rate of 95.1%. The sample incorporates 84 freshmen (25.4%), 130 sophomores (39.3%), 102 juniors (30.8%), 5 seniors (1.5%), and 10 graduate students (3.0%). Recruitment procedures, materials, and measures were first approved by Clark University’s Institutional Review Board, then all participants were recruited on campus between 2014 and 2015 and invited to take an online survey by the first author, who explained the study and procedure. The respondents voluntarily completed the questionnaire on their own time and without monitoring. After consenting to participate, respondents wrote down their answers to five questions and were allowed to write as much as they liked. Participants were entered into a random drawing for gift cards with a value equivalent to USD 10, with each participant having a 10% chance of receiving a gift card.

### 2.2. Measures

As noted, data were collected using a self-reported open-ended statement questionnaire, administered as part of a larger multinational study on purpose in higher education [47,48] (pp. 367–369). We collected responses to five question prompts at the beginning of each semester. Among these five questions, three were used to investigate the content of students’ life purpose, namely: what do you think is your life purpose, or the closest thing you have to a life purpose? why do you want to accomplish this life purpose? and what makes this life purpose important to you? The other two questions concerned the fulfillment of students’ life purpose: “describe any plans you have to act on your life purpose in the future; and describe any actions you are taking right now to accomplish your purpose”. This sequence of questions approximates those asked in clinical style interviews, which have been used to assess purpose in other studies [49]. Interviews are considered the gold standard purpose measure because the researcher can use follow-up and clarifying questions that effectively capture deep psychological experiences such as individuals’ purpose in life [50,51] (pp. 47–71). Our short answer questions were designed to imitate the interviews through progressive reflective questions that build on each other; thus, the information gathered is rich enough to look within and across participants’ responses, and to assign students to profiles based on their whole case and not just solitary questions.

### 2.3. Analysis

Data collection and analysis occurred in two phases. In phase one, and in discussion with colleagues in four other countries, we collected an initial set of student responses to the open-ended questions about students’ purposes in life, and developed a set of reliable codes on these pilot data based on themes that emerged from the responses [52]. Code development was the result of multiple discussions over time among students enrolled in ideological and political education classes, all of whom were masters and doctoral level students in that area. Further, they discussed codes with partner researchers and research teams both across countries and within China, including translation and back-translation to arrive at concepts that accurately captured the Chinese notion of purpose. These partner researchers and teams had online and in-person training in qualitative coding, and were upper-level graduate students or faculty with knowledge of purpose development. This process ultimately resulted in sets of well-defined codes that were validated and reliable in each country and language, and that were grounded in the locally collected data. Consequently, a Chinese codebook of responses was developed that could be used to analyze subsequent response data [28] (pp. 526–531). We then collected the response data among the participants described in the present research, applied the codes to their responses by engaging a team of coders who were education faculty members or graduate students in China, and calculated statistics to gauge code reliability (Cohen’s Kappa). 

Table 1 gives examples of statements representing each code. Coders applied codes of (1) “mentioned”, (2) “described”, or (3) “storied” to each response to the questions about purpose content and fulfillment. These codes captured how specific students were about their purpose, with greater specificity suggesting more elaborate thinking about purpose in their lives. The “mentioned” code was assigned to responses in which participants wrote about a specific focus, behavior, plan, or reason related to their purpose, but only at a general level and without reference to descriptive adjectives. In these examples, a purpose may be mentioned but not explained or discussed. The “described” code was given to responses with more detailed information about the purpose than was available in the mentioned responses, such as explanation of when, where, how, or who pertaining to the respondent’s purpose. The “storied” code was assigned to statements in which the respondent elaborates more fully on their purpose, such as providing logical explanations about how they envision things happening in their lives related to their purpose or which include language reflecting causality and temporal relationships pertaining to their purpose. If respondents did not specify a purpose or if statements were vague (i.e., statements such as “I don’t know” or “I’m not sure”) then the statement was coded as (0) “non-specific”. A fourth code, “beyond-the-selfness” was applied to student responses about why they want to accomplish their purpose, and what makes their purpose important to them. Responses were coded as (1) or present when the statement showed evidence that the respondent intends that their purpose would benefit others and not just themselves, or as (0) or absent, when statements indicated only self as the beneficiary. Thus, a code of purpose beyond-the-self represents answers related to the reason and importance of life purpose as it includes a beyond-the-self attribute [53] (pp. 412–439). Final reliability statistics were assessed for each 25% (*κ* = 0.55), 50% (*κ* = 0.78), 75% (*κ* = 0.80), and 100% (*κ* = 0.89) of the data, and indicated consensual agreement. In short, the process resulted in codes assigned to each response for each participant, thus providing opportunity for the researchers to use the code assignments to conduct further analysis in a variety of ways. For instance, researchers could examine code tag occurrence by question across the full sample, by question within each subject, or across questions within each subject. This latter approach constitutes a holistic, full-case approach through which researchers can then examine emergent themes to identify and validate potential profiles, and it is possible by this method to also search for emergent themes across the whole cases. In the next phase, the research team used this holistic full-case approach to identify qualitative purpose profiles among participants.

Using participants’ whole-case responses as the unit of analysis, purpose profiles were identified and defined in phase two using selected features of practices recommended in Consensual Qualitative Research [54] (pp. 1277–1288). CQR is derived from constructivism and incorporates some post-positivist elements [54] (pp. 1277–1288). It seeks to infer phenomena from a number of subjects, relying on their language expressions and the background information of the whole case, seeking to reach a consensus between researchers during the analysis processes [55] (pp. 466–485). CQR also emphasizes applying unified procedures and measures across subjects, as our study did, to enhance functionality and emphasizes the classification of subjective experience and behavioral responses. Our use of CQR analysis methods included (1) using a team to arrive at consensus judgments for profile definition and allocation, (2) using auditors to check work, (3) coding for domains and then extracting core ideas from these domains, and (4) systematically comparing the data across cases [56] (pp. 517–572).

First, a new group of six coders comprising doctoral and masters students read responses of each case in order to further organize the data into units including domains, core ideas, and categories. In this process, the data were first divided into several domains. Initial themes were tentatively inferred from existing theories and previous research results with consideration of the current data. Each researcher read the responses of the cases independently and grouped the data into different topics. After completing several cases, the team members discussed the division of each record and revised the topic until agreement was reached. After that, the stage of compiling core ideas was initiated. Each member of the team independently made a brief summary for each case under the theme of each paragraph, and again discussed their conclusions to reach an agreement. 

Within-case analysis was followed by a cross-case analysis, which was an attempt to extract what categories could be generated under each profile. In this way, we assigned cases to different profiles based on their content, which means all cases under each profile were gathered together. Group members repetitively read and discussed initial conclusions, and then classified feasible categories and sub-categories from them based on their understanding of all the data. Throughout each stage of the processes described here, results were checked by two auditors, who were professors with substantial knowledge of the study, offering the opportunity for timely feedback and suggestions. This included ensuring that coders returned to the original data to verify the rationality and authenticity of the conclusions.

## 3. Results

Four purpose profiles were identified among the study participants, namely, beyond-the-self purpose (*N =* 180, 54.4%), self-oriented life goal (*N =* 142, 42.9%), daydreamer (*N =* 6, 1.8%), and purposeless (*N =* 3, 0.9%). For some of these profiles, qualitatively different sub-groups also emerged. Table 2 lists brief descriptions and occurrences of each profile. The profiles are then explained in more depth, with statement examples rendered in English.

### 3.1. Beyond-the-Self Purpose

When expressing their reasons for and the importance of their life purpose, student teachers who discussed any entity other than the self as beneficiaries were recognized as having an altruistic purpose in life (coded as “purpose beyond-the self” being present). As explained earlier, the beyond-the-self (BTS) attribute of purpose mainly indicated that participants involved impacting entities other than themselves (i.e., imparting positive benefits) when they responded to questions about the reasons or importance of their purpose. These entities included families, friends, their students, a certain field, society, the country, or even the world. Further analysis showed that people with altruistic purpose repeatedly emphasized the benefits to other entities when they discussed the content, reasons for, and importance of their life purpose. In addition, coders found interesting differences between participants in this category, resulting in three sub-categories: active BTS purpose, less active BTS purpose, and action-directed BTS purpose. These subcategories are differentiated based on the clarity of the life purpose and on the engagement and planning related to pursuit and achievement of the purpose. They are described in more detail below. 

### 3.2. Active Beyond-the-Self Purpose (N = 127, 38.4%)

Participants identified within the active BTS purpose profile made statements that were coded as “mentioned”, “described” or “storied” on both reason/importance and engagement of life purpose along with “purpose beyond-the-selfness” for answers to reason/importance questions (see Materials and Methods). Participants in this category had very explicit ideas about their ultimate goals in life, and their ultimate goals shared the common feature of altruistic concerns, though this was sometimes in concert with self-related aims. When they discussed their purpose in life, they expressed a clear understanding of their mission for being in the world, striving for the well-being and benefit of others. Student teachers in this category also had very specific plans for how to achieve their purposes and were acting upon these plans directed toward their ultimate aims in life. This active engagement differentiates them from the less active BTS purpose group. 

In summary, student teachers coded as members of this group had a clear understanding of BTS-oriented purposes and had planned for and were actively pursuing those plans. This may indicate that student teachers in this sub-category have a mature cognitive understanding of their purpose and have reached a level of behavioral maturity, leading them to engage in relevant activities actively [57] (pp. 1–13). Thus, the active BTS purpose sub-category is seen as the most mature state of BTS purpose development in this study. Below is an example of statements written by a student teacher from this sub-category as they describe their efforts to positively influence students, which is their ultimate aim: 

“I am searching for my ideals in life. As a teacher, my life should be able to make a lot of changes to others. In this sense, in my life I hope to constantly improve myself, update my thinking, and maximize the positive impact on my students. I want to have a happy family and a healthy body, which are also close to my life ideals. The reason I chose this purpose is multiple. My life purpose comes from the university I attended and my clear future career as a teacher, with a combination of what I read from books and various media. Making changes among my students is important to me because human beings have their emotions. And every individual has the value of his/her own existence in life. This ideal of mine will help me achieve the value of my existence. At the same time, life purpose related to my self exists because human life occurs only once. We must not only think of others, but also be able to find our own happiness in connection with external things.” (Participant 5135; coded as “storied” purpose and “purpose beyond-the-self” present). 

This quotation demonstrates that the participant had a comprehensive understanding of what makes their life meaningful and valuable: exerting positive influence on their students and bringing changes to their lives. The same participant responded to questions about purpose plans and actions (engagement) as follows: 

“The activities I am taking now are the same as those I personally plan to take in order to achieve my ideals in life. I want my plans to be consistent with my current situation, and they should be. I’m reading many books and enriching my mind. I am participating in voluntary teaching services to improve my teaching skills, communication skills with students, and the ability to better understand students. Also, I’m also participating in various clubs to exercise my impromptu speech ability and organizational skills. I’m participating in many lectures and I made my own career plan. Now, I’m trying my best to study hard and gain enough expertise. I always consult my teachers about their suggestions on how I can improve myself.” (Participant 5135; coded as “storied” purpose). 

Participant 5135 was a typical example in this sub-category. Beyond having a clear BTS purpose in life, they had explicit plans about how to realize that purpose and is currently acting upon these plans. In this sense, they are already on the way towards achieving their ultimate goal in life. Though all our participants are student teachers, their purposes vary widely due to a differing personal understanding of meaning in life and their respective choices. Another example from this sub-category illustrates an entirely different purpose, but also explicitly discussed the ultimate aim of benefiting people in the world with very specific plans and actions. The following statements were written in response to the three questions about purpose content:

“The ideal of life is to use one’s strength and the creation of computer science and software to influence others and change people’s way of life in the future. It’s not a world of information that can’t be opened or peered into from the outside, as Steve Jobs described, but a world where we can provide others with a platform that we can create. Now the development of the Internet and computer science just provides such an opportunity to make the world a better place, letting human beings live in harmony with nature. Change some people’s thinking mode. Change the world with the Internet, and conquer people’s hearts with logic and happiness. I work hard step by step and start with a program. Because I want to see a world where everyone can give full play to their abilities, a world of invention and creation, a world of progress, a world based on cyberspace, a world where virtual and reality are integrated, and a world where everyone can create without being shackled by books.” (Participant 5588; coded as “storied” purpose and “purpose beyond-the-self” present).

In making the following statements, participant 5588 then explained their plans and actions (purpose engagement): 

“I plan to learn professional knowledge well. Besides, I learn more coding knowledge in extracurricular classes. I plan to practice first with the school website, and gradually improve the rules and regulations of the studio in charge. I plan to do a good job on the website of the students’ affairs office and the school website, deal with the relationship between studio members, divide work fairly and correctly and be a good person in charge. While learning from senior schoolmates, I also plan to help the lower class-men to learn, helping them making progress together and doing a better job together. I’m striving for more opportunities for exercise and internships for my classmates. At the beginning of the next semester, I plan to publicize the studio among new comers of this university.

After graduating from this University, I plan to find a good job, and strive for the opportunity to study abroad, so that I can learn the latest and best coding knowledge. And then I’ll start to build my desired cyberspace.” (coded as “storied” purpose). 

### 3.3. Less Active Beyond-the-Self Purpose (N = 31, 9.4%)

Participant profiles of less active BTS purpose wrote statements about the reason and importance of their purpose that were coded as either “mentioned”, “described”, or “storied” and were also coded as “purpose beyond-the-selfness”. Their statements about their plans and actions pertaining to their purpose were coded as “mentioned”. These codes indicate that student teachers who report a less active BTS purpose had BTS aims, such as those in the active BTS purpose sub-category, and their understanding of the reasons and importance of their BTS life purpose was rather clear and complex. However, engagement of their life purpose, although present, includes only simple plans and actions. Therefore, their purpose mainly manifested at the cognitive level and was lacking detailed plans and corresponding actions in their lives. These participants showed high levels of BTS-oriented intention, but lower levels of engagement. An example of this sub-category is demonstrated in the following response to questions about purpose content (what, why, and importance):

“I want happiness and freedom in my life. My purpose is becoming a teacher and making my own contribution to society. This is because of my own experience. I met a very good teacher when I was in middle school. This teacher made me understand that education was very important to children. It makes a lot of sense for me to recognize that educating children and cultivating them to become the future of our country is such a great thing.” (Participant 5645; coded as “described” purpose and “purpose beyond-the-self” present).

Yet, when asked the questions about plans and actions, participant 5645 wrote: “Study hard to build a solid foundation; Accumulate experience and make contributions to the teaching career.” (coded as “mentioned”). The above example illustrates that participant 5645 had a clear understanding of an ultimate far-reaching goal for their life, which exhibited a BTS reason for helping their future students by offering them good education, so that they were able to contribute to the bright future of the country. Yet, the plan and actions they mentioned were quite general, without specific plans articulated beyond vague expressions that are applicable to any student teacher.

### 3.4. Action-Guiding Beyond-the-Self Purpose (N = 22, 6.6%)

In this group, codes of “mentioned”, “described” and “purpose beyond-the-self” (present) were assigned to statements to questions related to the reasons and importance of purpose, and codes of “described” and “storied” were assigned to responses that answered the questions related to purpose plans and action. Participants with action-guiding BTS purpose showed BTS concern when discussing their purpose. The most distinctive characteristic of participants in this sub-category is that their purposes are largely plan and action oriented, with less clear intentions apparent. Thus, they may mention their purpose, the reasons for this purpose and its personal importance, but describe their plans and engagement in more detail. This means that their life purpose is described in a relatively general way, despite high involvement in planning and action related to that purpose. This makes the action-guiding BTS sub-category distinct from those mentioned above, in that participants in this profile seem to have action rather than intention driving their pursuit of a purpose. Thus, through their actions they may be clarifying or confirming their commitment to their purpose. A good example of this profile type is illustrated by the following quotation, arising in response to questions about the content of one’s purpose (what, why, and importance):

“I want to be a favorite teacher who can help students. My childhood experience in rural areas made me realize the gap in education between urban and rural areas. I want to narrow the gap. I want to influence others, and contribute to China’s education. I hope China’s education can be in the forefront of the world.” (Participant 5540; coded as “described” and “purpose beyond-the-self” present).

We can see from this statement that this participant described a future life through narrative in regard to how reducing rural–urban educational divides was personally meaningful and valuable. In this, this participant expressed that this purpose entailed working to address this inequality. Regarding specific actions and plans, this participant responded: 

“My general direction is to enter a high-quality high school and become a teacher there through hard work. I’ll expand my influence through working hard there. And then I will start to teach in rural areas. In my junior year, I will learn English in addition to the professional courses, and learn to play another instrument, such as the zither. I’ll find a desirable job in my senior year. In the first ten years after I start working, I will have worked hard to improve my professional skills and accumulate my influence in this profession. Within 20 years after I start working, I’ll have made an influence on rural education. And after that, I will continue to devote myself wholeheartedly to rural education.” (Participant 5540; coded as “storied” purpose).

These responses are typical of this subcategory. When asked about achieving their purpose, participants in this sub-category expressed complex and detailed plans and aims. However, this is in contrast to the less elaborate reasons and meanings they express in speaking about their intentions. Here, while these participants have been coded as having a BTS purpose, their statements do not seem to be as qualitatively intricate, involved, or elaborate in this purpose as active BTS purpose participants, largely because of this less complex intentionality.

### 3.5. Self-Oriented Life Goal

If student teachers’ responses tended to express self-involvement in the absence of a focus on others, they were classified under the profile of self-oriented (SO) life goal. Participants in this group are primarily pursuing intentions with regard to benefits to the self, rather than those that involve others in their statements to explain the reasons for or importance of their purpose. These individuals seem to be largely concerned with their own affairs and to have plans and actions related to the achievement of these goals. The data illustrated that student teachers with SO life goals pursue their own interests and benefits psychologically, materially, or both, as the most important pursuits in their lives. Self-satisfaction is an important indicator for these statements in judging whether or not students in these profiles feel they have achieved their goals. The expression of what they see as their life purpose reinforces their sense of the self as important. One hundred and forty-two participants were coded into this category, with three sub-categories, related to the categories for BTS purpose: active self-oriented life goal, less active self-oriented life goal, and action-directed self-oriented life goal.

### 3.6. Active Self-Oriented Life Goal (N = 70, 21.1%)

Participants with active SO life goals had similar coding and descriptive features to those with active BTS purpose, except that their statements were coded as “purpose beyond-the-self” absent. Student teachers in this sub-category clearly articulated their life goals and were actively pursuing detailed plans to make progress toward those goals. They strived to satisfy their own needs and to pursue their own good with motivation and determination. Here is an example of a quotation from a student teacher in this sub-category, responding to prompts regarding what their purpose was, why that was their purpose, and why it is important:

“I want to have a stable job with passion instead of boredom. What I want to do most is to travel around the world in my spare time, and visit the world’s famous mountains and rivers and places of interest. I want to see the scenery with my own eyes, and then write down some thoughts and what I think when I travel all over the world. It’s best to live in every place for a few months and experience the local customs with my heart. I want to feel life where the ancients left traces and purify my heart in the mountains and rivers. Maybe it’s because I like Chinese classical culture. I like to experience the joys and sorrows of ancient characters. I’m fond of walking, writing, yearning for a free and unrestrained life and pure mountains and forests.” (Participant 5400; coded as “storied” purpose).

In the above response, the participant explains their life goals explicitly and logically, demonstrating a clear understanding of what was most significant and meaningful in their life. The focus of attention is also self-oriented, emphasizing personal enjoyment and gratification. This participant, like others in this subcategory, focused on personal feelings and interests, aiming to achieve personal happiness. In response to questions about plans and actions relating to their purpose, the same participant wrote:

“Now, I will study hard and read more books on other aspects when I have time. I will also work hard for my purpose. I will strive to become an excellent lecturer to accomplish my own research. Meanwhile, I will also concentrate on my work in order to save enough money and time for myself, so that when I want to go out, I could go.” (Participant 5400; coded as “storied” purpose).

This participant is very clear about the kind of efforts they will make to pursue important life goals. This participant made specific plans regarding future work and research, but the ultimate reasons for doing so are to save time and money to allow for the SO pursuits of enjoying traveling and experiencing the world. This participant understood the importance of their life goals and oriented their pursuits to emphasize possibilities for traveling. Thus, even work plans are directed toward the possibility of traveling when not working. This pattern is typical of those in the active SO life goal sub-category.

### 3.7. Less Active Self-Oriented Life Goal (N = 68, 20.5%)

Participants with less active SO life goals were also coded as “purpose beyond-the-self” absent on questions related to the reason for and importance of purpose. Similar to the active SO life goal sub-category, student teachers with less active SO life goals possess a clear and complex understanding of what they would strive for in their lives and why these strivings are important to them. However, they demonstrated a comparatively lower level of purpose engagement, with more general and superficial plans and actions, lacking detail when asked about purpose-related plans and actions. This combination of high intention amidst lower engagement contributed to less mature life goal pursuit for those in this sub-category as compared to the active SO life goal sub-category. We infer that the general developmental processes for these participants are in some ways similar to those in the less active BTS purpose sub-category. The following is an example of the kind of response given by participants in this category to questions about the content, reasons, and importance of their purpose:

“I hope that I can have a happy family in the future. In addition, I also want to improve my ability at present to have a job with high salary and high position in my future. At the same time, I want to have a few close friends and we could go into politics together. This is how I understand my life ideals.” (Participant 5379; coded as “storied” purpose). 

When asked about his/her plans relating to this purpose, the same participant wrote: “I plan to work hard to learn professional knowledge and read more extracurricular books. I will participate in as many meaningful practical activities as possible.” (Participant 5379, coded as “mentioned” purpose).

This example illustrates that the participant regarded their stated life goals as a means to achieve personal meaning. Mention of friends in the future is largely described in terms of the benefits of having these friends for the self, not the benefits of their life goals for friends. The response to questions about future plans and actions were very general and routine, with little mention of current activity. In this, Participant 5379 is representative of others in this subcategory who have strong SO intentions, but did not clearly articulate complex plans for engagement with those intentions.

### 3.8. Action-Guiding Self-Oriented Life Goal (N = 4, 1.2%)

Participants in the action-guiding SO life goal sub-category expressed their SO intentions in comparatively general ways, when compared to those in the less active SO life goal and active SO life goal subgroups. They did, however, exhibit detailed plans and specific actions to achieve their life goals. Thus, they show greater evidence of engagement than intention. They are also similar, in many ways, to the action-guiding BTS purpose sub-category. The following quotation is an example of the ways that members of this sub-category describe the content, reasoning and meaning of their life goals:

“I want to be a chemistry teacher because I love chemistry and chemistry education. Also, I admire several chemistry teachers.” (Participant 5350; coded as “described” purpose).

This demonstrates relatively simple and unelaborated reasoning for wanting to be a chemistry teacher compared to those in other sub-categories of SO life goal. Little detail is provided about reasons for a love of chemistry or the reasons for admiring teachers. The same participant had much more elaboration with regard to plans and actions:

“My major is chemistry at a normal (teacher) university. I plan to try my best to study chemistry knowledge at college and constantly review chemistry knowledge I learned at middle school. I will continue to be a volunteer teacher in the chemistry group until I leave this University. Later, I plan to take a master’s degree in education and be a chemistry tutor. Finally, I plan to be a chemistry teacher at a middle school.” (Participant 5350; coded as “storied” purpose). 

The above statements demonstrate that this participant had clear and elaborate plans about upcoming years and post-collegiate life. Like others in the action-direct SO life goal sub-category, this participant has a clear plan for achieving their most personally important life goals and was actively engaged in pursuing these goals. However, participants in this sub-category are less articulate about their purpose than the active SO life goal sub-category, as the reasons for and meaning of these pursuits are less elaborated and distinct.

### 3.9. Daydreamers

The third profile, which we labeled daydreamers, includes two subgroups, one exhibiting BTS oriented dreams (*N* = 1, 0.3%), the other with SO dreams (*N* = 5, 1.5%). These participants clearly discussed answers to questions about the content (what), reasons, and importance of their purpose. Those in this profile, however, did not report any plans or actions concerning their purpose or goals. This means that these individuals show evidence of having thought about meanings and goals, but lacked the behavioral motivation or a sense of pathways to engage in relevant activity pertaining to their stated purposes. Below is an example of statements of BTS-oriented purposes written by participant 5126:

“I want to be filial to my parents and be busy in my career and family because I must return the kindness of parents. Also, I want to open my mind to new things and keep learning in order to living out of myself to complete the current study and future improvement.” (Participant 5126; coded as “described”). No plans or actions were mentioned in the answer. 

Participant 5216’s responses to questions about reason/importance were coded as self-oriented purpose: “I want to achieve individual values to get spiritual satisfaction of myself.” (Participant 5216; coded as “described”). Here, again, no further plans or actions were provided in the answer. 

Daydreamers like this are more closely related to the less active BTS purpose and SO life goal sub-categories than to the others above, though their lack of activity and engagement means that they appear less mature than those sub-categories.

### 3.10. Purposeless

The final profile identified in this dataset is those student teachers who had little to say in answering the questions about what their purpose is and why that purpose is personally important. Further, these individuals gave little detail regarding plans or actions related to these reported purposes. As such, they were coded as purposeless. These individuals seem to be engaged in various activities, but without considering the relationship between those activities and personal meanings and goals. Their descriptions of plans and actions were also brief, indicating relatively simple plans without explicit and elaborated ideas about how they wanted to pursue a meaningful life. For example, Participant 5184 only responded to questions about plans and actions (engagement) concerning purpose as follows:

“I will study hard and enrich myself.” (Participant 5184; coded as “mentioned”). No purpose was provided or elaborated in the purpose what/why/importance parts. This category, as it lacks clear intention or engagement, seems to be the least articulate category of participants in the sample as pertains to explaining a purpose for their lives.

## 4. Discussion

This study examines student teachers’ responses to inquiries about their purpose in life, within a Chinese context. The results suggest that Chinese student teachers could be divided into four broad categories based on their expressions of the content, reason for and importance of their perceived purpose, and plans and actions relating to that purpose. These four profiles are BTS purpose, SO life goal, daydreamers, and the purposeless. The first two categories comprise 95% of cases, which is a solid majority of the sample. These two categories were also subdivided into three parallel subcategories: active, less active, and action-driven BTS purpose and SO life goals. 

These findings are different from those of prior studies, with a greater proportion pf participants in the sample categorized as BTS purpose than has been found in large scale interview studies; we found over 70% to constitute BTS profile cases as compared to roughly 25–30% in other studies [11,13] (pp. 715–733; pp. 143–159). Studies based on fewer questions derived directly from longer interviews to identify purpose have found larger proportions, rising to nearly 40% [42] (pp. 1–24). One might conclude that the present sample is highly purposeful, in that the vast majority can identify their purpose, such that student teachers may be a special population with higher levels of purpose development than other youth. This possibility is in line with some prior evidence [19] (pp. 186–199), though the level may also be driven by the relative clarity of and reasons supported by participants’ career choice in education, the open-ended statement method, the cultural context, or the coding criteria of this study. 

In this study, the most distinctive difference between the two largest categories, BTS purpose and SO life goal, lay in the actual intended beneficiaries of the goal. For the BTS purpose group, participants showed distinct BTS or prosocial orientation in describing the intended beneficiaries or consequences of their pursuits. For BTS purposeful youth, their future students, their families and friends, society, and country were intended beneficiaries of their purposeful pursuits. In contrast, participants with SO life goals expressed their goals as primarily benefiting themselves, through personal achievement, gratification, or meaning. In concrete terms, this means that BTS purposeful youth think about the reasons or importance of their pursuit of purpose as contributing to the betterment of future students, society, or reducing social inequalities, while youth with SO life goals emphasize reasons regarding time, money, amusement, and personal status.

The three sub-categories of BTS purpose and SO life goals are also important to more fully consider. According to one theory of mature purpose [57] (pp. 1–13), these profiles may constitute developmental steps on the way to full active BTS purpose, which manifests all three key components of purpose: intention, engagement, and BTS orientation [13] (pp. 143–159). This theory has still to be further validated empirically, such as in longitudinal studies, and it includes additional components than those included here. Nonetheless, there is some logic to the idea that mature functioning includes the capability of exhibiting and coordinating all three features of purpose across and within one’s life. This is to say that less active BTS purpose is intermediate between what Damon [10] (pp. 59–61) called dreaming and active purpose, manifesting some engagement, but not mature engagement, just as action-driven BTS purpose is intermediate between dabbling and active purpose, manifesting some intention but not mature intention. Similar claims can be made with regard to SO life goals, with less active SO life goals and action-driven SO life goals as potential developmental precursors to more mature SO life goals. Further, Reilly and Mariano [57] (pp. 1–13) would suggest that BTS purpose is more mature than SO life goals, as implied by the three key components of purpose. 

This leaves two further categories, daydreamers and the purposeless. Daydreamers were those who expressed their understanding of their ultimate goals in life, including both BTS-oriented and SO manifestations. These student teachers were similar to Damon’s [10] (p. 59) dreamer profile, though SO daydreamers, the most common form in this sample, lack the BTS orientation of Damon’s dreamer profile. The purposeless, lacking clear personal goals and so unable to relate actions to those goals, could be akin to either Damon’s dabbler or drifter profiles. However, among the present sample, these categories were quite small, representing, across daydreamers and the purposeless, roughly 3% of the sample. This may be a result of the age, culture, and context of the participants, as the participants are older than many of those in Damon’s [10] (pp. 52–53) study and in normal college. This is also unlike the more educationally diverse young adults in Glanzer and colleagues’ [11] (pp. 715–733) study, which found roughly 30% in the directionless category, which was most similar to the purposeless category in the present study.

This research provides preliminary evidence of varied purpose profiles among student teachers in China. Further, this analysis highlights purpose profiles on a continuum, with subcategories not present in prior research, such as inactive BTS purpose and action-guiding BTS purpose, rather than merely noting the presence or absence of purpose. Based on data analysis, this sample showed evidence of greater maturity than those in other research, based both on the high rate of active BTS purpose, higher rates of other subcategories of BTS purpose, and lower rates of daydreamers and purposeless than in other studies. As such, we anticipate that this group is more likely to be developing optimally, in line with other studies of purpose development [19,30] (pp. 186–199; p. 52). 

### Limitations and Future Directions

In this study, data collection about life purpose from student teachers was mainly based on the written content of answers to an online survey. This provided an opportunity to share the content, reasons for, importance of, plans, and actions related to their perceived purpose. However, this should be understood in comparison with the methods of other studies, as, for instance, no interviewer was able to prompt for elaboration with the online survey as they might with an online or in-person interview. Additionally, other samples of student teachers and those preparing for other professions would provide additional information regarding how broadly the findings of the present study generalize. For instance, is this sample similar to student teachers from other regions of China, or of students studying to enter business, politics, engineering, or health fields?

Future research would do well then to adopt different methods, engage with varied samples, test interventions, and to retain participants longitudinally. Different methods might help to understand the differences between experiences that contribute to the different sub-categories of BTS purpose and SO life goal found in this study and provide more efficient ways of assessing them (e.g., with surveys such as that of [51] (pp. 47–71), and others). Longitudinal research may clarify if it is the case that youth who have active BTS purpose prior to college are more likely to pursue education as a student teacher rather than some other educational goal. Additionally, it would be valuable to determine if student teachers’ expression of purpose becomes more mature over time, such that freshmen are more likely to be identified in the less mature categories, and the same individuals respond in more mature ways as juniors and seniors. Intervention research could add to this consideration of the specific experiences and processes that support or hinder the development of more mature purpose.

## 5. Conclusions

In summary, the present research helps to identify and articulate the general purpose profiles among Chinese student teachers. This helps to better understand this population, especially their understanding of meaning in life and their ideas about purpose. It demonstrates that the present sample has higher rates of BTS purpose than those found in other studies and presents three sub-categories of BTS purpose, relating these categories to prior research. This research also highlights possibilities for purpose education and interventions, as it demonstrates that many student teachers remain uncertain of their purposes. 

## Figures and Tables

**Table 1:** Examples of statements coded for purpose specificity and beyond-the-selfness.

Code	Example
**Purpose Specificity**	
Mentioned	“I plan to work hard to learn professional knowledge and read more extracurricular books. I will participate in as many meaningful practical activities as possible.” (case 5379, physics education, female, 20 years old, sophomore)
Described	“I want happiness and freedom in my life. My purpose is becoming a teacher and making my own contribution to society. This is because of my own experience. I met a very good teacher when I was in middle school. This teacher made me understand that education was very important to children. It makes a lot of sense for me to recognize that educating children and cultivating them to become the future of our country is such a great thing.” (case 5645, arts education, female, 21 years old, junior)
Storied	“I plan to learn professional knowledge well. Besides, I learn more coding knowledge in extracurricular classes. I plan to practice first with the school website, and gradually improve the rules and regulations of the studio in charge. I plan to do a good job on the website of the students’ affairs office and the school website, deal with the relationship between studio members, divide work fairly and correctly and be a good person in charge. While learning from senior schoolmates, I also plan to help the lower class-men to learn, helping them making progress together and doing a better job together. I’m striving for more opportunities for exercise and internships for my classmates. At the beginning of the next semester, I plan to publicize the studio among new comers of this university. After graduating from this University, I plan to find a good job, and strive for the opportunity to study abroad, so that I can learn the latest and best coding knowledge. And then I’ll start to build my desired cyberspace.”(case 5588, physical education, female, 20 years old, sophomore)
**Purpose Beyond-the-Selfness**	
Aim includes benefit to others	“I want to be a favorite teacher who can help students. My childhood experience in rural areas made me realize the gap in education between urban and rural areas. I want to narrow the gap. I want to influence others, and contribute to China’s education. I hope China’s education can be in the forefront of the world.” (case 5540, chemistry education, male, 20 years old, sophomore)
Aim omits benefit to others	“I hope that I can have a happy family in the future. In addition, I also want to improve my ability at present to have a job with high salary and high position in my future. At the same time, I want to have a few close friends and we could go into politics together. This is how I understand my life ideals.” (case 5379 physics education, female, 20 years old, sophomore)

**Table 2:** Occurrence of qualitative purpose profiles.

Profile	Frequency	Percent
**Beyond-the-self purpose**	**180**	**54.4%**
*Active*	127	38.4%
*Less active*	31	9.4%
*Action-guiding*	22	6.6%
**Self-oriented life goal**	**142**	**42.9%**
*Active*	70	21.1%
*Less active*	68	20.5%
*Action-guiding*	4	1.2%
**Daydreamer**	**6**	**1.8%**
*BTS-oriented*	1	0.3%
*Self-goal*	5	1.5%
**Purposeless**	**3**	**0.9%**
***Total***	**331**	**100%**

## Data Availability

The datasets for this study can be found in the Center for Ideological and Political Education, Northeast Normal University. Further inquiries can be directed to the first author.
